# Reporting Bias Is Highly Prevalent in Systematic Reviews and Meta-Analyses Related to Medial Patellofemoral Ligament Reconstruction

**DOI:** 10.1016/j.asmr.2025.101213

**Published:** 2025-07-03

**Authors:** Kenneth T. Nguyen, Erin L. Brown, William C. Rittmeyer, Shreya M. Saraf, Mia V. Rumps, Mary K. Mulcahey

**Affiliations:** a Tulane University School of Medicine, New Orleans, Louisiana, U.S.A.; b Department of Orthopedic Surgery and Rehabilitation, Loyola University Medical Center, Maywood, Illinois, U.S.A.

## Abstract

**Purpose:**

To analyze reporting bias in the form of spin present in systematic reviews and meta-analyses related to medial patellofemoral ligament reconstruction (MPFLR).

**Methods:**

Following the Preferred Reporting Items for Systematic Reviews and Meta-Analyses guidelines, systematic reviews were collected from PubMed, Web of Science, and Embase using the search “medial patellofemoral ligament reconstruction” or “MPFLR” AND “systematic review” OR “meta-analysis.” Abstracts were assessed for 15 common spin types. A Measurement Tool to Assess Systematic Reviews 2 (AMSTAR 2) was used to evaluate the quality of the studies. Characteristics such as Preferred Reporting Items for Systematic Reviews and Meta-Analyses adherence, publication year, and level of evidence were analyzed. Associations between these factors and spin presence or type were determined using statistical tests, including *t* tests, analysis of variance, Fisher tests, and Spearman rank coefficients.

**Results:**

The initial database search identified 1,044 studies, of which a total of 57 studies were included. Spin was present in the abstract in 51 of 57 studies (89.5%). Each type of spin was observed in at least 1 study’s abstract with the exceptions of spin types 1, 7, 13, and 15. The 3 most common types were type 5 (48/57, 84.2%), followed by type 3 (32/57, 56.1%) and type 9 (30/57, 52.6%). Of the included studies, 91.2% received a critically low AMSTAR 2 confidence rating, and only 5.3% reported a conflict of interest. There was a statistically significant negative correlation between the numerical AMSTAR 2 rating and the presence of spin (*P* < .01).

**Conclusions:**

Most systematic reviews on MPFLR received critically low AMSTAR 2 ratings, reflecting the poor quality of evidence in this area. Nearly 90% of abstracts exhibited at least 1 type of spin, with spin types 3, 5, and 9 being the most common, suggesting a tendency to overstate the efficacy of MPFLR for patellar instability.

**Level of Evidence:**

Level IV, systematic review of Level II-IV studies.

Medial patellofemoral ligament reconstruction (MPFLR) is commonly performed to treat recurrent patellar instability.^1^ To ensure that the results of MPFLR are accurately portrayed in the literature and are of high quality, it is important to review published studies for reporting bias in the form of spin. Spin is defined as a “specific way of reporting, intentional or not, to highlight that the beneficial effect of the experimental treatment in terms of efficacy or safety is greater than that shown by the results (i.e., overstate efficacy and/or understate harm).”^2^

By examining the degree of spin within a study, the precision and accuracy with which findings are reported in the literature can be determined. The assessment of spin scrutinizes research at the level of results reporting. According to Yavchitz et al.,^2^ spin can be organized into 3 different categories: misleading representation, misleading reporting, and inappropriate extrapolation. Furthermore, prior studies by Lazarus et al.^3^ and Boutron et al.^4^ have shown that spin is a frequent occurrence in abstracts, appearing in 58.3% of randomized controlled trials with nonsignificant primary outcomes and 84% of nonrandomized intervention studies, respectively. These findings underscore how commonly spin can shape first impressions of research findings, even in otherwise methodologically sound studies. Physicians rely heavily on abstracts as their primary source of information for making therapeutic decisions, potentially influencing the perceived effectiveness of an intervention when spin is present.^5^, ^6^, ^7^

Although prior studies have evaluated spin in systematic reviews across various orthopaedic procedures, MPFLR remains understudied in this context. The body of literature surrounding MPFLR is relatively diverse and continues to expand, making it important to assess how these studies are being summarized in abstracts. These characteristics may increase susceptibility to spin in abstracts, as authors attempt to reconcile heterogeneous findings. The purpose of this study was to analyze reporting bias in the form of spin present in systematic reviews and meta-analyses related to MPFLR. We hypothesized that spin would be commonly present in abstracts of systematic reviews and meta-analyses related to MPFLR, reflecting trends previously reported in the broader clinical research literature, and that greater levels of spin would be associated with lower methodologic quality.

## Methods

This study was conducted according to the Preferred Reporting Items for Systematic Reviews and Meta-Analyses (PRISMA) guidelines using a predetermined protocol. The methodology, including eligibility criteria, data extraction strategy, and spin classification approach, was established prior to the conduct of the review to ensure consistency and minimize bias in the assessment process. Two authors (K.T.N. and E.L.B.) conducted a search of the PubMed, Web of Science, and Embase databases using “medial patellofemoral ligament reconstruction” or “MPFLR” AND “systematic review” OR “meta-analysis” in January 2024. The search results were aggregated and deduplicated in Covidence. Two authors (K.T.N. and E.L.B.) independently screened the identified studies for inclusion, while the third author (W.C.R.) resolved remaining conflicts.

### Eligibility

Systematic reviews and meta-analyses related to MPFLR published in an English peer-reviewed journal were eligible for inclusion. Databases were queried from inception to January 22, 2024. Studies were excluded if they were not peer-reviewed, were not published in English, were not systematic reviews and/or meta-analyses, were retracted or withdrawn, included nonhuman or cadaver subjects, were unrelated to MPFLR, were published without an abstract, or did not have full text available.

### Training

Three authors (K.T.N., E.L.B., and W.C.R.) received training on spin and the AMSTAR 2 grading system by independently reviewing relevant literature on both topics, as well as in the definition and classification of the most common types of spin proposed by Yavchitz et al.,^2^ as summarized in Table 1.^8^^,^^9^ The authors also learned to assess study quality using A Measurement Tool to Assess Systematic Reviews 2 (AMSTAR 2).^10^ The adoption of AMSTAR 2 for assessing study quality is supported by its impressive inter-rater reliability and high construct validity.^11^

**Table 1:** Description of Types of Spin Assessed

Category	Type	Description
Misleading interpretation		
	1	The conclusion formulates recommendations for clinical practice not supported by the findings.
	2	The title claims or suggests a beneficial effect of the experimental intervention not supported by the findings.
	4	The conclusion claims safety based on nonstatistically significant results with a wide confidence interval.
	9	The conclusion claims the beneficial effect of the experimental treatment despite reporting bias.
	12	The conclusion claims equivalence or comparable effectiveness for nonstatistically significant results with a wide confidence interval.
Misleading reporting		
	3	Selective reporting of or overemphasis on efficacy outcomes or analysis favoring the beneficial effect of the experimental intervention.
	5	The conclusion claims the beneficial effect of the experimental treatment despite a high risk of bias in primary studies.
	6	Selective reporting of or overemphasis on harm outcomes or analysis favoring the safety of the experimental intervention.
	10	Authors hide or do not present any conflict of interest.
	11	The conclusion focuses selectively on a statistically significant efficacy outcome.
	13	Failure to specify the direction of the effect when it favors the control intervention.
	14	Failure to report a wide confidence interval of estimates.
Inappropriate extrapolation		
	7	The conclusion extrapolates the review findings to a different intervention (e.g., claiming efficacy of one specific intervention, although the review covered a class of several interventions).
	8	The conclusion extrapolates the review’s findings from a surrogate marker or a specific outcome to the global improvement of the disease.
	15	The conclusion extrapolates the review’s findings to a different population or setting.

Using AMSTAR 2, a 16-question critical appraisal tool, studies were graded by each author (K.T.N., E.L.B., and W.C.R.) on their methodologic quality and assigned a confidence rating. This tool evaluates an author’s incorporation of a predetermined study protocol, funding source, conflicts of interest, and authors’ overall ability to adequately characterize the findings of studies included in the review. The full texts of the included studies were used to assess study quality per the AMSTAR 2 checklist, which yielded a numeric score between 0 and 16. This score reflects the extent to which the studies meet quality standards, with the numerical value representing the number of AMSTAR 2 checklist elements found in the study. In addition to the numerical score, the AMSTAR 2 critical appraisal tool distinguishes between deficiencies in critical and noncritical domains. In doing so, the AMSTAR 2 assessment identifies critical flaws in systematic reviews and meta-analyses by assigning studies critically low, low, moderate, and high confidence ratings.^2^^,^^10^

### Data Extraction

Three authors extracted data independently (K.T.N., E.L.B., and W.C.R). In the case of disagreement, resolution was achieved after discussion between the 2 authors (K.T.N. and E.L.B.) or input from a third author (W.C.R.). Study characteristics from the full text that were extracted included title, authors, publication year, study design, journal, funding source, level of evidence, reported adherence to PRISMA guidelines, preregistration status, and outcome measures. If not stated within the study, the level of evidence was determined using the American Academy of Orthopaedic Surgeons recommendations.

The full text of each study was assessed for the 15 most common types of spin (Table 1).^2^ For studies reporting confidence intervals, an interval was considered wide if it spanned more than 2 units, included both sides of the null value, or exceeded 50% of the point estimate in relative width. The full texts of the included systematic reviews were used to assess study quality per the AMSTAR 2 checklist, which yielded a numeric score between 0 and 16. The AMSTAR 2 confidence ratings were also extracted. Additionally, the impact factor was recorded for the journals in which the included systematic reviews and meta-analyses were published. This metric, which gauges the significance of a journal by totaling the number of citations its selected articles receive over a recent period (typically the past few years), serves as a valuable tool for comparing journals within a specific subject category. A higher impact factor indicates a more esteemed journal.

### Data Analysis

Descriptive statistics were used to characterize the frequency of spin occurring in the included studies. Study characteristics, including study type, level of evidence, funding source, PRISMA adherence, PROSPERO registration, impact factor, and AMSTAR 2 confidence rating, were analyzed. Their association with the presence of spin, as well as the number of spin types present, was determined using *t* tests, analysis of variance, Fisher tests, and Spearman rank coefficients. A *P* value <.05 was considered statistically significant.

## Results

**Fig 1:**
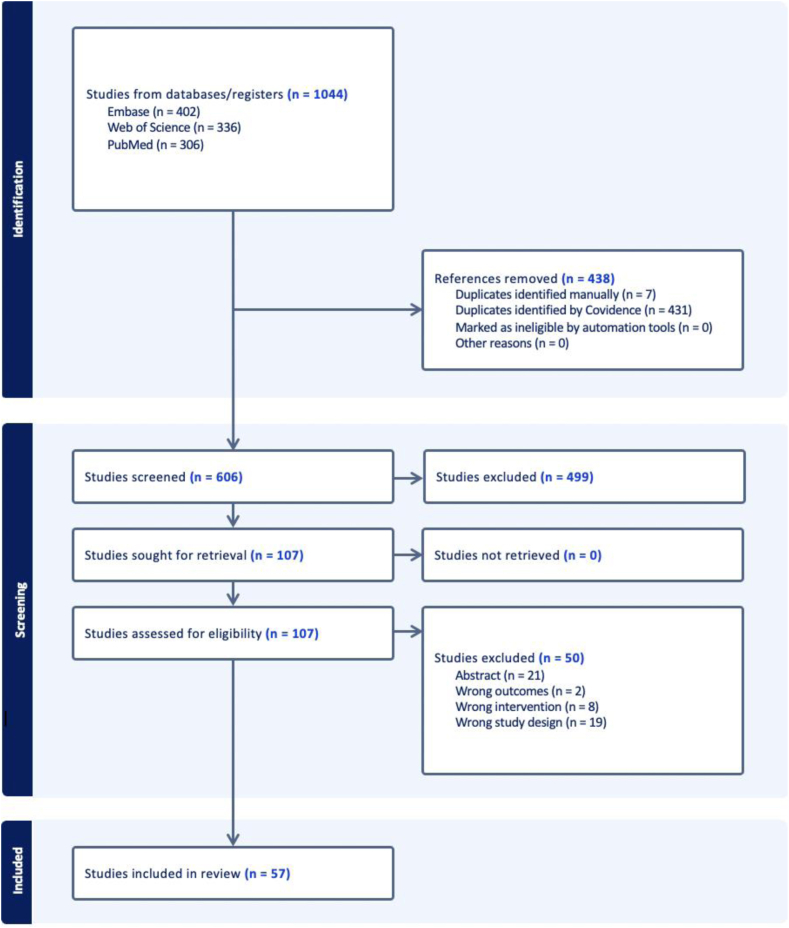
Preferred Reporting Items for Systematic Reviews and Meta-Analyses (PRISMA) flow diagram.

The initial database search identified 1,044 studies, of which 438 duplicates were removed. An additional 499 studies were removed after title and abstract screening because they did not meet the inclusion criteria. The remaining 107 studies were assessed for eligibility. Of the 107, 50 (46.7%) were excluded because they were abstracts only (21, 42.0%), had the wrong study design (19, 38.0%), investigated the wrong intervention (8, 16.0%), or identified the wrong outcomes (2, 4.0%) (Fig 1). The 57 (53.3%) remaining systematic reviews, which were published in 24 different journals with dates of publication ranging from 2010 to 2024, were included for analysis.^12^, ^13^, ^14^, ^15^, ^16^, ^17^, ^18^, ^19^, ^20^, ^21^, ^22^, ^23^, ^24^, ^25^, ^26^, ^27^, ^28^, ^29^, ^30^, ^31^, ^32^, ^33^, ^34^, ^35^, ^36^, ^37^, ^38^, ^39^, ^40^, ^41^, ^42^, ^43^, ^44^, ^45^, ^46^, ^47^, ^48^, ^49^, ^50^, ^51^, ^52^, ^53^, ^54^, ^55^, ^56^, ^57^, ^58^, ^59^, ^60^, ^61^, ^62^, ^63^, ^64^, ^65^, ^66^, ^67^, ^68^

Most of the included studies were Level of Evidence IV (46 out of 57, 80.7%), 6 (10.5%) were Level of Evidence III, 4 (7.0%) were Level of Evidence II, and 1 (1.8%) was Level of Evidence V. Twenty-nine studies (50.9%) included a systematic review with meta-analysis, while 28 studies (49.1%) were systematic reviews without meta-analyses. Twelve studies (21.1%) did not disclose funding, 16 (28.1%) disclosed at least 1 external source of funding, and 29 studies (50.9%) received no funding. Three studies (5.3%) submitted their protocols to the PROSPERO public register of systematic reviews. Almost all studies reported PRISMA adherence (52/57, 91.2%). Twenty-four different journals were represented among these systematic reviews, for which the mean impact factor was 2.95 ± 2.33 (range, 0.5-12.2) (Table 2).

**Table 2:** Study Characteristics of Included Systematic Reviews and Meta-Analyses

Author	Year Published	Journal	Impact Factor	Level of Evidence	PRISMA Adherence	PROSPERO Preregistration
Abbaszadeh et al.^12^	2023	*World Journal of Clinical Cases*	1.1	IV	Yes	No
Abelleyra Lastoria et al.^13^	2023	*Knee Surgery & Related Research*	3.1	IV	Yes	No
Aicale and Maffulli^14^	2020	*Journal of Orthopaedic Surgery and Research*	2.7	IV	Yes	No
Aliberti et al.^15^	2021	*Orthopaedic Journal of Sports Medicine*	2.8	IV	Yes	No
Almeida et al.^16^	2023	*Journal of Clinical Orthopaedics and Trauma*	3.3	IV	Yes	No
An et al.^17^	2019	*Journal of Orthopaedic Surgery*	1.8	III	Yes	No
Balcarek et al.^18^	2017	*KSSTA*	3.4	IV	No	No
Boutefnouche et al.^19^	2016	*KSRR*	1.8	IV	Yes	No
Buckens and Saris^20^	2010	*American Journal of Sports Medicine*	3.9	IV	No	No
Burnham et al.^21^	2016	*Journal of Arthroscopic and Related Surgery*	4.5	IV	No	No
Castagno et al.^22^	2023	*The Knee*	2.0	IV	Yes	No
Cohen et al.^23^	2022	*KSSTA*	4.2	IV	Yes	No
D’Ambrosi et al.^24^	2021	*Children*	2.6	IV	Yes	No
Fancher et al.^25^	2021	*Orthopaedic Journal of Sports Medicine*	2.8	IV	Yes	No
Fang et al.^26^	2023	*Frontiers in Surgery*	2.0	III	Yes	No
Fisher et al.^27^	2010	*Arthroscopy*	3.1	IV	Yes	No
Guevel et al.^28^	2023	*Frontiers in Surgery*	2.0	IV	Yes	No
Heo et al.^29^	2019	*American Journal of Sports Medicine*	6.0	IV	Yes	No
Hussein et al.^30^	2018	*Journal of ISAKOS*	1.9	II	Yes	No
Jiang et al.^31^	2023	*Indian Journal of Orthopaedics*	1.0	IV	Yes	No
Kahlon et al.^32^	2023	*KSSTA*	4.2	II	Yes	No
Kalinterakis et al.^33^	2023	*European Journal of Orthopaedic Surgery and Traumatology*	1.8	IV	Yes	No
Kang et al.^34^	2019	*KSSTA*	3.4	IV	Yes	No
Kang et al.^35^	2018	*Archives of Orthopaedic and Trauma Surgery*	2.3	IV	Yes	No
Liu et al.^36^	2021	*Orthopaedic Journal of Sports Medicine*	2.6	III	Yes	Yes
Mackay et al.^37^	2014	*Orthopaedic Journal of Sports Medicine*	2.6	IV	Yes	No
Manjunath et al.^38^	2021	*American Journal of Sports Medicine*	6.0	IV	Yes	No
MarínFermín et al.^39^	2022	*Journal of Orthopaedic Surgery and Research*	2.7	IV	Yes	No
Migliorini et al.^40^	2022	*Journal of Sport and Health Science*	13.0	IV	Yes	No
Migliorini et al.^41^	2020	*Archives of Orthopaedic and Trauma Surgery*	2.5	IV	Yes	No
Migliorini et al.^42^	2022	*Journal of Orthopaedics and Traumatology*	3.0	IV	Yes	No
Migliorini et al.^43^	2022	*Children*	2.6	IV	Yes	No
Migliorini et al.^44^	2023	*Children*	2.4	IV	Yes	No
Migliorini et al.^45^	2021	*Journal of Orthopaedic Surgery and Research*	2.7	IV	Yes	No
Migliorini et al.^46^	2020	*European Journal of Orthopaedic Surgery & Traumatology*	1.9	IV	Yes	No
Nha et al.^47^	2019	*KSRR*	1.6	V	Yes	No
Pang et al.^48^	2023	*American Journal of Sports Medicine*	6.1	III	Yes	No
Pappa et al.^49^	2023	*Journal of ISAKOS*	1.8	IV	Yes	No
Plat et al.^50^	2022	*American Journal of Sports Medicine*	6.1	IV	Yes	No
Ren et al.^51^	2019	*Archives of Orthopaedic and Trauma Surgery*	2.6	IV	Yes	No
Schneider et al.^52^	2016	*American Journal of Sports Medicine*	5.3	IV	Yes	No
Shamrock et al.^53^	2019	*Orthopaedic Journal of Sports Medicine*	2.7	IV	Yes	No
Singhal et al.^54^	2013	*The Bone & Joint Journal*	3.0	IV	No	No
Smith et al.^55^	2007	*KSSTA*	1.6	IV	Yes	No
Tan et al.^56^	2022	*European Journal of Orthopaedic Surgery & Traumatology*	1.8	IV	Yes	No
Tanos et al.^57^	2023	*Medical Sciences*	2.1	IV	Yes	No
Testa et al.^58^	2017	*KSSTA*	3.4	III	Yes	No
Ulrich et al.^59^	2020	*Journal of Arthroscopy and Joint Surgery*	0.6	IV	Yes	No
Vivekanantha et al.^60^	2023	*KSSTA*	4.3	IV	Yes	No
Walker et al.^61^	2022	*KSSTA*	4.3	IV	Yes	No
Wang et al.^62^	2024	*KSSTA*	4.3	IV	Yes	Yes
Weinberger et al.^63^	2017	*KSSTA*	3.4	IV	Yes	No
Wilkens et al.^64^	2020	*KSSTA*	3.4	IV	Yes	Yes
Xing et al.^65^	2020	*Journal of Orthopaedic Surgery and Research*	2.8	II	Yes	No
Xu et al.^66^	2023	*Orthopaedic Surgery*	2.3	IV	Yes	No
Yoo et al.^67^	2023	*Medicine*	1.8	II	Yes	No
Zhang et al.^68^	2020	*Orthopaedic Journal of Sports Medicine*	2.8	III	No	No

*Journal of ISAKOS*, *Journal of the International Society of Arthroscopy, Knee Surgery and Orthopaedic Sports* Traumatology; *KSRR*, *Knee Surgery & Related Research*; *KSSTA*, *Knee Surgery, Sports Traumatology, Arthroscopy*; PRISMA, Preferred Reporting Items for Systematic Reviews and Meta-Analyses.

Based on AMSTAR 2 assessment, 5 studies (8.8%) received a low confidence rating due to the presence of 1 critical flaw. The remaining 52 studies (91.2%) received a critically low confidence rating due to the presence of more than 1 critical flaw. No studies fell into the moderate or high confidence rating categories, both of which required the presence of zero critical flaws. Any discrepancy in AMSTAR 2 assessment between the authors was resolved with a discussion regarding semantics. The median AMSTAR 2 score was 10 (range, 5-14, mean = 9.46 ± 2.51) (Table 3).

**Table 3:** AMSTAR 2 Assessment of Reviewed Studies

AMSTAR 2 Question (N = 57)	Yes, n	%
1. Did the research questions and inclusion criteria for the review include the elements of PICO?	56	98.3%
2. Did the report of the review contain an explicit statement that the review methods were established prior to the conduct of the review and did the report justify any significant deviations from the protocol?∗	16	28.1%
3. Did the review authors explain their selection of the study designs for inclusion in the review?	24	42.1%
4. Did the review authors use a comprehensive literature search strategy?∗	56	98.3%
5. Did the review authors perform study selection in duplicate?	56	98.3%
6. Did the review authors perform data extraction in duplicate?	56	98.3%
7. Did the review authors provide a list of excluded studies and justify the exclusions?∗	5	8.8%
8. Did the review authors describe the included studies in adequate detail?	49	86.0%
9. Did the review authors use a satisfactory technique for assessing the risk of bias (RoB) in individual studies that were included in the review?	40	70.2%
10. Did the review authors report on the sources of funding for the studies included in the review?∗	0	0.00%
11∗. If meta-analysis was performed did the review authors use appropriate methods for statistical combination of results?	40	70.2%
12. If meta-analysis was performed, did the review authors assess the potential impact of RoB in individual studies on the results of the meta-analysis or other evidence synthesis?	17	29.8%
13. Did the review authors account for risk RoB in individual studies when interpreting/ discussing the results of the review?∗	29	50.9%
14. Did the review authors provide a satisfactory explanation for, and discussion of, any heterogeneity observed in the results of the review?	26	45.6%
15. If they performed quantitative synthesis did the review authors carry out an adequate investigation of publication bias (small study bias) and discuss its likely impact on the results of the review?∗	21	36.8%
16. Did the review authors report any potential sources of conflict of interest, including any funding they received for conducting the review?	47	82.5%

AMSTAR 2, A Measurement Tool to Assess Systematic Reviews 2; PICO, population, intervention, comparator group, and outcome.

∗ Critical domain.

### Frequency of Spin and Analysis

Spin was not identified in the abstracts of 6 of 57 (10.5%) studies. Spin was present in the abstracts of the other 51 studies (89.5%). Each type of spin was observed in at least 1 study, with the exceptions of spin types 1, 7, 13, and 15. The median number of spin types identified per study was 3 (range, 0-5, mean = 3.02 ± 1.45). The 3 most common types of spin were types 5 (48/57, 84.2%), 3 (32/57, 56.1%), and 9 (30/57, 52.6%) (Table 4). For example, 1 abstract reflected type 3 spin by claiming that surgical management of medial patellofemoral ligament reconstruction injuries led to better outcomes than conservative treatment, despite considerable heterogeneity in surgical technique and limited supporting evidence.^30^ Type 5 spin was exemplified by an abstract that concluded equivalent outcomes between single- and double-bundle MPFLR despite relying primarily on low-level case series.^34^ Type 9 spin was observed in an abstract that reported favorable outcomes for MPFLR without acknowledging the potential reporting bias introduced by unclear inclusion criteria and methodologic variability.^37^ The category of spin that was most prevalent was misleading reporting (spin types 3, 5, 6, 10, 11, 13, 14), present in 50 of the 51 studies (98.0%). Misleading reporting also had the highest frequency, with a total of 107 instances across the 50 studies. Forty-seven of 51 (92.2%) studies contained spin within the category of misleading interpretation (spin types 1, 2, 4, 9, 12). However, the frequency of misleading interpretation was lower than that of misleading reporting, with a total of 62 instances out of the 47 studies.

**Table 4:** Frequency of Spin Type and Category

Category	Type	Description	Abstracts, n (%)
Misleading interpretation			
	1	The conclusion formulates recommendations for clinical practice not supported by the findings.	0 (0.0%)
	2	The title claims or suggests a beneficial effect of the experimental intervention not supported by the findings.	1 (1.8%)
	4	The conclusion claims safety based on nonstatistically significant results with a wide confidence interval.	26 (45.6%)
	9	The conclusion claims the beneficial effect of the experimental treatment despite reporting bias.	30 (52.6%)
	12	The conclusion claims equivalence or comparable effectiveness for nonstatistically significant results with a wide confidence interval.	5 (8.8%)
Misleading reporting			
	3	Selective reporting of or overemphasis on efficacy outcomes or analysis favoring the beneficial effect of the experimental intervention.	32 (56.1%)
	5	The conclusion claims the beneficial effect of the experimental treatment despite a high risk of bias in primary studies.	48 (84.2%)
	6	Selective reporting of or overemphasis on harm outcomes or analysis favoring the safety of the experimental intervention.	5 (8.8%)
	10	Authors hide or do not present any conflict of interest.	13 (22.8%)
	11	The conclusion focuses selectively on a statistically significant efficacy outcome.	9 (15.8%)
	13	Failure to specify the direction of the effect when it favors the control intervention.	0 (0.0%)
	14	Failure to report a wide confidence interval of estimates.	6 (10.5%)
Inappropriate extrapolation			
	7	The conclusion extrapolates the review findings to a different intervention (e.g., claiming efficacy of one specific intervention, although the review covered a class of several interventions).	0 (0.0%)
	8	The conclusion extrapolates the review’s findings from a surrogate marker or a specific outcome to the global improvement of the disease.	1 (1.8%)
	15	The conclusion extrapolates the review’s findings to a different population or setting.	0 (0.0%)

There was not a statistically significant association between level of evidence and the presence of spin or the number of spin types present (*P* = .256 and *P* = .448). Similarly, there was not a statistically significant association between year of publication and the presence of spin or the number of spin types present (*P* = .999 and *P* = .673). The AMSTAR 2 assessment identifies critical flaws in systematic reviews and meta-analyses by assigning studies critically low, low, moderate, and high confidence ratings; this study found that AMSTAR 2 confidence rating was not significantly associated with the presence of spin.

However, the AMSTAR 2 confidence rating was significantly associated with the number of spin types present, meaning that a study with a critically low confidence rating was statistically more likely to have more spin types present when compared to the studies that had a low confidence rating (*P* < .001). There was also a statistically significant association between the numerical AMSTAR 2 rating and the presence of spin. The full text for the studies was used to assess study quality per the AMSTAR 2 checklist, which yielded a numeric score between 0 and 16. This score reflects the extent to which the studies meet quality standards, with the numerical value representing the number of AMSTAR 2 checklist elements found in the study. The average numerical AMSTAR 2 score for studies with no spin was 11.7 (range, 10-14), while the average for studies with spin was 9.2 (range, 5-14) (*P* < .001). As the number of spin types present increased, the AMSTAR 2 rating decreased in a statistically significant manner (*P* = .007).

There was no statistically significant association between the journal impact factor and the presence of spin. The average impact factor of the studies that had spin present was 3.15 ± 1.76, while the average impact factor of the studies that did not have spin present was 3.13 ± 0.78 (*P* = .986). As the number of spin types present increased, the impact factor of the journal in which the study was published tended to decrease. However, this finding was not statistically significant (*P* = .067).

## Discussion

This study underscores the frequent presence of spin and the predominance of low-quality evidence among MPFLR reviews, as assessed by AMSTAR 2, highlighting the need for more rigorous research practices and critical appraisal of existing literature. Of the 57 systematic reviews analyzed, 51 (89.5%) abstracts had evidence of at least 1 type of spin. Fifty-two (91.2%) systematic reviews had more than 1 significant methodologic error, according to concurrent AMSTAR 2 assessment, giving them a critically low confidence rating. Furthermore, none of the studies in our sample earned a grade of either moderate or high confidence. This shows the low quality of evidence for systematic reviews and meta-analyses related to MFPLR.

Multiple studies have assessed the types and prevalence of spin in orthopaedic literature, emphasizing how common it is. In their analysis of systematic reviews and meta-analyses pertaining to midshaft clavicle fractures, Gulbrandsen et al.^69^ discovered that spin was present in 52.8% of abstracts, with spin type 3 being the most prevalent (28.3%). Similarly, Thompson et al.^70^ examined spin in ulnar collateral ligament reconstruction (UCLR) systematic reviews and meta-analyses and found that all included studies had at least 1 type of spin. The most common type of spin was type 5 (“the conclusion claims the beneficial effect of the experimental treatment despite a high risk of bias in primary studies”), which accounted for 36.8% of the total. Kumaran et al.^71^ expanded this analysis to include systematic reviews and meta-analyses on lateral extra-articular tenodesis and anterolateral ligament reconstruction for anterior cruciate ligament (ACL) injuries. The authors found that spin type 9—which appears in 95.5% of studies—was the most prevalent (“conclusion claims the beneficial effect of the experimental treatment despite reporting bias”). In accordance with our study, spin types 3, 5, and 9 were most frequently found, with spin type 5 showing up in 84.2% of abstracts. Doyle et al.^72^ examined spin in systematic reviews on the remplissage procedure and found that 86.7% of abstracts included at least 1 form of spin, with type 9 being the most common (73%). Gulbrandsen et al.^69^ also studied spin in systematic reviews of quadriceps tendon grafts for ACL reconstruction and reported spin in 53.8% of abstracts, with type 3 as the most frequent (30.8%). Some of the variations in findings may be partially explained by differences in the characteristics of the studies included in each review. The number of articles analyzed, the types of interventions covered, and the way outcomes were reported could all influence how spin appears and is identified. In addition, although each study used predefined criteria for detecting spin, small variations in how raters interpret and apply these definitions may contribute to differences in reported prevalence and spin types. Even with standardized tools, subtle differences in judgment are difficult to eliminate entirely. Collectively, these studies highlight how spin frequently downplays or conceals methodological errors to highlight the effectiveness or safety of surgical procedures such as midshaft clavicle fracture operations, UCLR, ACL reconstruction, anterolateral ligament reconstruction, lateral extra-articular tenodesis, or MPFLR. This highlights a broader trend in orthopaedic research toward overstating positive conclusions, underscoring the need for critical appraisal by readers to mitigate the impact of bias on clinical decision-making.

Interestingly, while spin was present in research published by both high-impact and low-impact journals, our analysis did not find a significant correlation between the journal impact factor and spin. Similarly, in systematic reviews of UCLR, Thompson et al.^70^ found no consistent relationship between impact factor and spin prevalence, but they did discover that studies that did not employ validated risk-of-bias techniques were more likely to be published in lower-impact journals. On the other hand, studies without spin had a much higher chance of being published in higher-impact journals and receiving more citations, according to Gulbrandsen et al.^69^ Together, these findings highlight that while journal impact factor alone may not reliably predict the presence of spin, there is a clear association between spin and lower methodologic quality. This underscores the importance of critical appraisal to ensure that conclusions, particularly in abstracts, accurately reflect the underlying evidence and maintain scientific integrity.

Evaluation of spin is a crucial tool for identifying bias in the reporting of results, particularly in systematic reviews and meta-analyses, which aim to provide comprehensive summaries of evidence. While sound study design and tools like the Cochrane Risk of Bias tool are vital for minimizing methodologic flaws, spin can undermine objectivity and mislead readers, even in rigorously conducted studies. As supported by multiple studies across various orthopaedic procedures, including MPFLR, hip arthroscopy, and ligament reconstructions, spin-filled abstracts often overemphasize favorable outcomes, obscure limitations, and create unwarranted confidence in clinical interventions.^69^, ^70^, ^71^ This concern is amplified by the reality that abstracts are frequently the only section read by clinicians—especially in settings where access to full-text articles is limited. When selective reporting or statistical embellishment is present, it can distort the interpretation of nonsignificant results and prematurely shape clinical practice.^73^

In the context of MPFLR, spin in systematic reviews and meta-analyses is particularly relevant, as these studies are intended to provide the most complete and reliable summaries of evidence. According to Boutron et al.^74^ and Marcelo et al.,^7^ spin-filled abstracts can cause orthopaedic surgeons to misinterpret results or create favorable attitudes about surgical procedures prematurely. Manuscripts containing promotional abstracts must be critically evaluated and thoroughly read to verify that the treatment intervention is backed by sufficient and reliable evidence to ensure that MPFLR is used effectively.^26^^,^^44^^,^^50^

### Limitations

There are several limitations to this study. First, identifying spin is a subjective process, despite the measures taken to avoid bias, including a simultaneous collection process by 3 reviewers. Second, this study was limited by the low level of evidence utilized by most of the included studies; most were of Level IV, with no Level I and few Level II studies. Additionally, there were intrinsic limits to certain study characteristics. For example, many studies were published prior to the release of PRISMA guidelines. It is unclear when journals started endorsing PRISMA criteria. Finally, the AMSTAR 2 instrument that we employed for evaluation was created and released in 2017. As a result, using it to evaluate systematic reviews that were published earlier in 2017 would have led to lower results.

## Conclusions

Most systematic reviews on MPFLR received critically low AMSTAR 2 ratings, reflecting the poor quality of evidence in this area. Nearly 90% of abstracts exhibited at least 1 type of spin, with spin types 3, 5, and 9 being the most common, suggesting a tendency to overstate the efficacy of MPFLR for patellar instability.

## Disclosures

The authors declare the following financial interests/personal relationships which may be considered as potential competing interests: M.K.M. serves on the boards or committees of the American Academy of Orthopaedic Surgeons, American Orthopaedic Association, American Orthopaedic Society for Sports Medicine, Arthroscopy Association of North America, Association of Bone and Joint Surgeons, and ISAKOS, among others, and holds editorial positions with the *American Journal of Sports Medicine*, *Arthroscopy*, *JBJS*, and *Ortho Info*. All other authors (K.T.N., E.L.B., W.C.R., S.M.S., M.V.R.) declare that they have no known competing financial interests or personal relationships that could have appeared to influence the work reported in this paper.
